# Zinc Oxide Tetrapods Modulate Wound Healing and Cytokine Release In Vitro—A New Antiproliferative Substance in Glaucoma Filtering Surgery

**DOI:** 10.3390/life12111691

**Published:** 2022-10-24

**Authors:** Svenja Rebecca Sonntag, Stefanie Gniesmer, Anna Gapeeva, Rainer Adelung, Ala Cojocaru, Yogendra Kumar Mishra, Sören Kaps, Aysegül Tura, Swaantje Grisanti, Salvatore Grisanti, Khaled Nassar

**Affiliations:** 1 Department of Ophthalmology, University of Lübeck, 23538 Lubeck, Germany; 2 Institute for Materials Science, Christian-Albrechts-University of Kiel, 24118 Kiel, Germany; 3 Phi-Stone AG, 24143 Kiel, Germany; 4 Mads Clausen Institute, NanoSYD, University of Southern Denmark, 6400 Sonderborg, Denmark

**Keywords:** glaucoma filtering surgery 1, nanotechnology 2, ZnO tetrapods 3, postoperative encapsulation 3, antiproliferative substances 4

## Abstract

Glaucoma filtering surgery is applied to reduce intraocular pressure (IOP) in cases of uncontrolled glaucoma. However, postoperative fibrosis reduces the long-term success of both standard trabeculectomy and microstents. The aim of this study was to test the antiproliferative and anti-inflammatory potential of ZnO-tetrapods (ZnO-T) on human Tenon’s fibroblasts (HTFs) for glaucoma surgery. The toxicity of ZnO-T on HTFs was determined using an MTT test. For analysis of fibroblast proliferation, migration, and transdifferentiation, cultures were stained for Ki67, alpha-smooth muscle actin (α-SMA), and *p*-SMAD. A fully quantitative multiplex ELISA was used to determine the concentrations of different cytokines, platelet-derived growth factor (PDGF), and hepatocyte growth factor (HGF) in culture supernatants with and without previous ZnO-T treatment. Treatment with higher concentrations (10 and 20 µg/mL) was associated with HTF toxicity, as shown in the wound healing assay. Furthermore, the number of Ki67, α-SMA-positive, and pSMAD-positive cells, as well as IL-6 and HGF in supernatants, were significantly reduced following incubation with ZnO-T. In conclusion, we were able to show the antiproliferative and anti-inflammatory potentials of ZnO-T. Therefore, the use of ZnO-T may provide a new approach to reducing postoperative fibrosis in glaucoma filtering surgery.

## 1. Introduction

Glaucoma, despite numerous advances in therapeutic options, is still the second most frequent cause of bilateral blindness worldwide. A recent review by Tham et al. states that there has been an increase in glaucoma diagnoses from 64.3 million people in 2013 to 76 million people in 2020 worldwide, which will further increase to 111.8 million people in 2040 [1]. According to Quigley et al., the number of bilateral open-angle glaucoma blindness cases also increased from 4.5 million people in 2010 to 5.9 million people in 2020 [2]. Still, the reduction of IOP is the most successful parameter in preventing glaucoma progression [3]. In addition to drug therapy, surgical interventions play a major role, especially in cases of inadequate drug IOP reduction or drug intolerance.

The success of glaucoma filtering surgeries is limited by postoperative encapsulation. The introduction of microstents has not reduced postoperative fibrosis, and five years after implanting glaucoma drainage devices, the success rate is only 40 to 50% [4]. Therefore, to reduce postoperative fibrosis, it is essential to influence the natural wound healing mechanisms.

Wound healing involves a complex series of interactions between cells, chemical signals, extracellular matrix (ECM) proteins, and microenvironments [4]. Proliferation, migration, and transdifferentiation of fibroblasts into myofibroblasts are crucial steps in wound healing processes [5]. Myofibroblasts provide mechanical support and integrity to the tissue after an injury [6]. Under physiological conditions, they are removed via apoptosis when the tissue integrity is sufficiently restored to be mechanically coherent [7]. In pathological wound healing processes, myofibroblasts persist in the tissue and are responsible for fibrosis via increased matrix synthesis and contraction of the tissue [8]. Therefore, modulation of fibroblast functions would improve the therapeutic options for diseases involving fibrosis [9].

The fibroblast cells targeted for the prevention of postoperative encapsulation are human Tenon fibroblasts (HTFs). HTFs, which are located below the conjunctiva [10], play an important role in the processes of scarring after glaucoma filtering surgery as well as in wound healing [11,12]. There are numerous studies investigating the exact mechanisms leading to postoperative scarring. It seems that not only growth factors but also increased oxidative stress play a major role in this process [13,14]. It could also be shown that in glaucoma patients, growth factors like TGF-beta are increased. TGF-beta can exert its profibrotic effect through the activation of IL-6. Furthermore, IL-6 itself and IL-1 seem to be able to stimulate the transdifferentiation of fibroblasts into myofibroblasts and therefore support scarring [15,16].

Those scarring mechanisms are also promoted by the fibrotic and inflammatory processes of local glaucoma therapy. Thus it is especially in this group of patients that scarring of the bleb after glaucoma surgery can occur more easily [17].

Antiproliferative substances such as mitomycin c or 5-fluorouracil are already used to counteract fibrous encapsulation [18]. Nevertheless, the IOP level often rises again postoperatively. Therefore, coating the drainage devices with antiproliferative drugs was tried to avoid postoperative fibrosis [19]. Still, the application of mitomycin c or 5-fluorouracil is not without risk. Late-onset leak (>3 months after surgery) is described and leads to bleb ischemia and breakdown of the conjunctiva, followed by postoperative hypotony and its complications [20]. Furthermore, both antiproliferative substances can cause corneal epithelial toxicity [18,21]. Various materials have been investigated to allow slow and controlled delivery of the antiproliferative substances mitomycin c and 5-fluorouracil, thereby reducing the postoperative complication rate [22,23]. Although the results have been promising, these delivery systems have not yet been used in patient care.

Newer studies focus on the possibility of combining coated drainage devices with nanotechnologies for optimized drug delivery [24]. Nanomaterials exhibit unique properties that differ from those at the macroscopic scale, e.g., they offer more sites for chemical reactions [25]. Ye et al. developed cationic nanocopolymers to reduce scarring and increase bleb survival [26]. Another study was able to address activated Tenon’s fibroblasts using MMC-incorporated LDL nanoparticles as the LDL receptor is over-expressed after the glaucoma filtering surgery in those cells [27]. Those studies show the potential of nanotechnology in glaucoma surgery.

Zinc Oxide Nanoparticles (ZnO NPs) are some of the most widely used nanomaterials in biomedicine [28]. Several reports confirm the antibacterial and virustatic effects of ZnO NPs [29,30,31]. Furthermore, ZnO NPs induce cytotoxicity [32] and apoptosis in dermal fibroblasts [33] and cancer cells [34].

ZnO tetrapods (ZnO-T) represent a new form of ZnO NPs that can be synthesized in large amounts with dimensions ranging from nanoscale to microscale [35]. 

The aim of this study was to evaluate the antiproliferative effects of ZnO-T on human Tenon’s fibroblasts (HTF) using immunohistochemistry and in vitro models of wound healing to find a new and innovative antiproliferative nanoparticle for glaucoma filtering surgery.

## 2. Materials and Methods

### 2.1. Preparation of Nano ZnO Tetrapods (ZnO-T)

Powder of ZnO-T was synthesized and investigated as described in a previous work [36]. The powder of ZnO-T was scaled and dispersed into culture medium at 1 mg/mL at the start of each experiment. One reproducible standard batch of ZnO-T served as reference in all experiments and was set in relation to other samples. As ZnO-T particles precipitate quickly, the dispersion was shaken by a vortexer (Werner Hassa GmbH, Luebeck, Germany) before a serial dilution (1 to 5000 µg/mL) was prepared in culture medium.

### 2.2. Scanning Electron Microscopy (SEM)

Micrographs of ZnO-T particles were obtained using the SEM microscope Zeiss Ultra Plus with the Gemini column (Carl Zeiss Microscopy GmbH, Jena, Germany) at 5 kV acceleration voltage.

### 2.3. ZnO-T Absorption Spectrum

A microplate spectrophotometer (SpectraMax M4, Molecular Devices, Sunnyvale, USA) was used to evaluate the absorption spectrum of the ZnO-T at 570 nm. Concentrations of 0, 0.1, 1, 10, 100, and 1000 µg/mL were tested.

### 2.4. Establishment of the Human Tenon’s Fibroblast (HTF) Cultures

Samples of the human Tenon’s capsule were obtained from patients undergoing cataract surgery. The study was in accordance with the tenets of the Declaration of Helsinki for the use of human tissue, and informed consent was obtained from the patients after explanation of the nature and possible consequences of the study. The generation of HTF cultures was performed as described previously [37]. Briefly, the tissue was dissected into 1- to 2-mm cubes and maintained in DMEM/F-12 (1:1) medium supplemented with 10% heat-inactivated fetal calf serum (FCS, Invitrogen-Gibco Life Technologies, Karlsruhe, Germany), 2 mM L-glutamine, 100 U/mL penicillin, and 100 µg/mL streptomycin (Biochrom, Berlin, Germany) in a 100-mm Petri dish at 37 °C in a humidified atmosphere with 5% CO_2_. The fibroblasts migrating from these tissues were harvested after approximately 3 weeks using incubation with 0.05% trypsin and 0.02% EDTA (Invitrogen), centrifuged at 300 *g* for 8 min, and seeded in fresh culture medium in 75 cm^2^ flasks. The cells between the third and ninth passages were used for the experiments.

### 2.5. Cell Viability after Incubation with ZnO-Tetrapods

HTFs were seeded at 5 × 10^3^ cells/well (n = 6) in 96-well plates and grown to confluence for 36 h. The ZnO-T stock solution was diluted in DMEM and added to the cell culture at different concentrations (0–15 µg, 20–200 µg, and 1000–5000 μg/mL). HTFs (p8) were grown initially for 36 h. The incubation period with the ZnO-T was 48 h. An MTT test was done as described [28], and the absorbance at 570 nm was measured using a microplate reader (Tecan Group Ltd., Maennedorf, Switzerland). The standard error of the mean (SEM) of three independent tests was calculated.

### 2.6. Ki67, α-SMA and pSMAD Immunostaining

HTFs were seeded in triplicates at a density of 3 × 10^4^ cells on each side of an Ibidi culture insert for live cell analysis (Ibidi, Munich, Germany) with a 500 μM separation between each side of the well and allowed to grow for 24 h. The cells were treated with different concentrations of ZnO-T (0, 2, 4, 6, 8, and 10 µg/mL). The treatment was ended after 6 h when a complete culture medium exchange was done. Forty-eight hours later, the cells were fixed in 2% paraformaldehyde (PFA) followed by 4% PFA for 10 min. Immunostaining was performed as described previously [37], using primary antibodies against Ki67 (dilution 1:300, MAB4190, Millipore, Hessen, Germany), alpha-smooth muscle actin (α-SMA) (dilution 1:100, Ab7817, Abcam, Cambridge, UK) or pSMAD 2 and 3 (1µg/mL, ab65847, Abcam, Cambridge, UK) followed by Alexa 488-conjugated anti-rabbit antibodies (diluted 1:100 in blocking buffer; Jackson Immuno-Research, Hamburg, Germany; Molecular Probes, Darmstadt, Germany, respectively). Nuclei were counterstained with DAPI (1 µg/mL in PBS) for 10 min. Stained HTFs were examined with an inverted microscope (Leica DMI 6000 B, Wetzlar, Germany). Photographs were captured using a DFC 290 compatible camera and the appropriate software (Leica Application Suite LAS Software, Wetzlar, Germany).

### 2.7. Quantification of Immunopositive cells

Immunopositive cells were counted using ImageJ software [38]. A grid was projected, the images were initialized, and the cell counter function was activated. The mean ± SEM of positive cells was calculated.

### 2.8. Cell Migration: Wound Healing Assay

HTFs were seeded at a density of 3 × 10^4^ cells on each side of an Ibidi culture insert for live cell analysis (Ibidi, Munich, Germany), with a 500 μM separation between each side of the well, and allowed to grow for 24 h. The cells were treated with different concentrations of ZnO-T (0, 1, 5, 10, and 20 µg/mL). The treatment was ended after 6, 24, or 48 h, when a complete culture medium exchange was done. 

Mosaic phase contrast microphotographs were captured using the Leica DMI 6000 B microscope and Leica Application Suite LAS Software (Leica Microsystems GmbH, Wetzlar, Germany). The use of the automated mosaic image capture allowed a complete assessment of the wound gap and prevented missing or overlapping certain areas. Images were imported to NIH ImageJ software, and the wound gap was calculated at 0, 24, and 48 h. To take into account lost cells at the wound rim, the unhealed area was compared to the double wound gap area. The rate of wound healing was calculated using the following equation:

(1) $$\mathrm{Rate}\;\mathrm{of}\;\mathrm{wound}\;\mathrm{healing}\;\mathrm{at}\;48\;h\;=\left(1-\frac{\mathrm{Unhealed}\;\mathrm{wound}\;\mathrm{area}\;\mathrm{at}\;48\;h}{\mathrm{Total}\;\mathrm{wound}\;\mathrm{area}\;\mathrm{at}\;0\;h\;\times \;2}\right)\;\times \;100$$

### 2.9. Culture Supernatant Samples

HTF culture supernatant samples were collected after incubation with ZnO-T (1, 5, 10, and 20 µg/mL) for 24 or 48 h. The control group was incubated without the ZnO-T. Samples were aliquoted under sterile conditions at volumes of 50 µL, labeled, and stored at –80 °C until use. 

### 2.10. Immunoassays

To evaluate the effect of ZnO-T on the inflammatory and wound healing characteristics of HTFs, samples were examined for 5 cytokines: IL (interleukin) -1α), IL-1 β, IL-6, platelet-derived growth factor (PDGF), and hepatocyte growth factor (HGF). Therefore, a customized fully quantitative multiplex ELISA (Q-Plex™ Human Cytokine arrays, Quansys Biosciences, Logan, UT, USA) that worked as a sandwich immunoassay was used. Each of the 5 spots within each well contained a distinct capture antibody population. The cytokine in each sample bound to its distinct capture antibody spots and subsequently to cytokine-specific, horseradish peroxidase (HRP)-bound secondary antibodies. Samples were tested using a high-sensitivity protocol. Samples were diluted at 1:2 and 1:5 in a Quansys human sample dilution buffer (Quansys Biosciences, Logan, UT, USA). Diluted standards and samples were added to wells containing 5-plex arrays and incubated on a plate shaker for 1 h at room temperature. The wells were then washed 3 times with washing buffer. A detection mix was added and incubated on a plate shaker for 1 h at room temperature. The wells were washed 3 times, then streptavidin–horseradish peroxidase (HRP) was added for 15 min, followed by 6 washes, and a substrate for the detection of the cytokines was added. To capture the biomarker concentration in each sample, an image of the plate was taken by the Quansys Q-view imager system (Quansys Biosciences, Logan, UT, USA).

### 2.11. Statistics

Statistical analysis was performed with GraphPad Prism 6 software for Windows (San Diego, CA, USA). The variable distribution was evaluated with the one-sample Kolmogorov–Smirnov test. Here the null hypothesis was defined as the data coming from a normal distribution. Significant results (*p* value <0.05) reject the null hypothesis (in such cases, a non-parametric test was selected for further analysis), while a non-significant test (*p* value > 0.5) confirmed the null hypothesis (i.e., a normal distribution of the data) and therefore the use of a parametric test for further analysis was appropriate. 

A one-way ANOVA test was used for observation with parametric distribution with matched follow-up values. A Kruskal–Wallis test was used for observation with non-parametric distribution and unmatched follow-up values. Dunn’s correction of a multiple comparison test using statistical hypothesis testing was applied as recommended by the software. 

Two-way ANOVA and Bonferroni post hoc tests were used to evaluate the in vitro experiment. Family-wise alpha threshold and confidence level were set to 0.5% and 95%, respectively. *p*-values less than 0.05 were considered statistically significant. Bonferroni post hoc test correction of multiple comparison tests using statistical hypothesis testing was applied as recommended by the software.

To estimate the half maximal inhibitory concentration (IC50), a 4-parameter logistic, nonlinear regression model was used (GraphPad Prism 6 software for Windows, San Diego, CA, USA)). All experiments were done in triplicates.

## 3. Results

### 3.1. Scanning Electron Microscopy (SEM)

**Figure 1:**
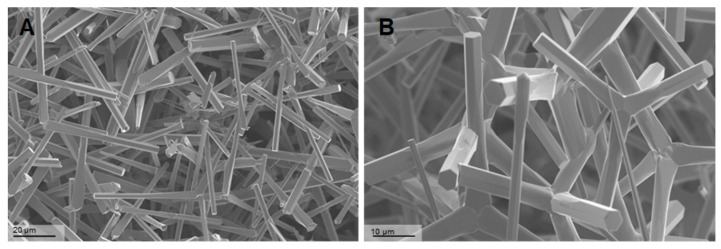
Scanning electron microscopy (SEM) of Zinc Oxide Tetrapods (ZnO-T): SEM photomicrographs of ZnO-T at different magnifications 708× (**A**) and 1.44K× (**B**)**,** showing the unique structure of the tetrapods. The arms of the tetrapods exhibit a hexagonal wurtzite crystal structure oriented along the c-axis, with alternating Zn^2+^ and O^2−^ stacking planes. The tetrapods have a tetrahedral geometry with 109.5 u bond angles to each other.

The thicknesses of the tetrapod arms varied from 500 nm to 5 µm. Their lengths ranged from 10 µm to 50 µm (Figure 1).

### 3.2. ZnO-T Absorption Spectrum

**Figure 2:**
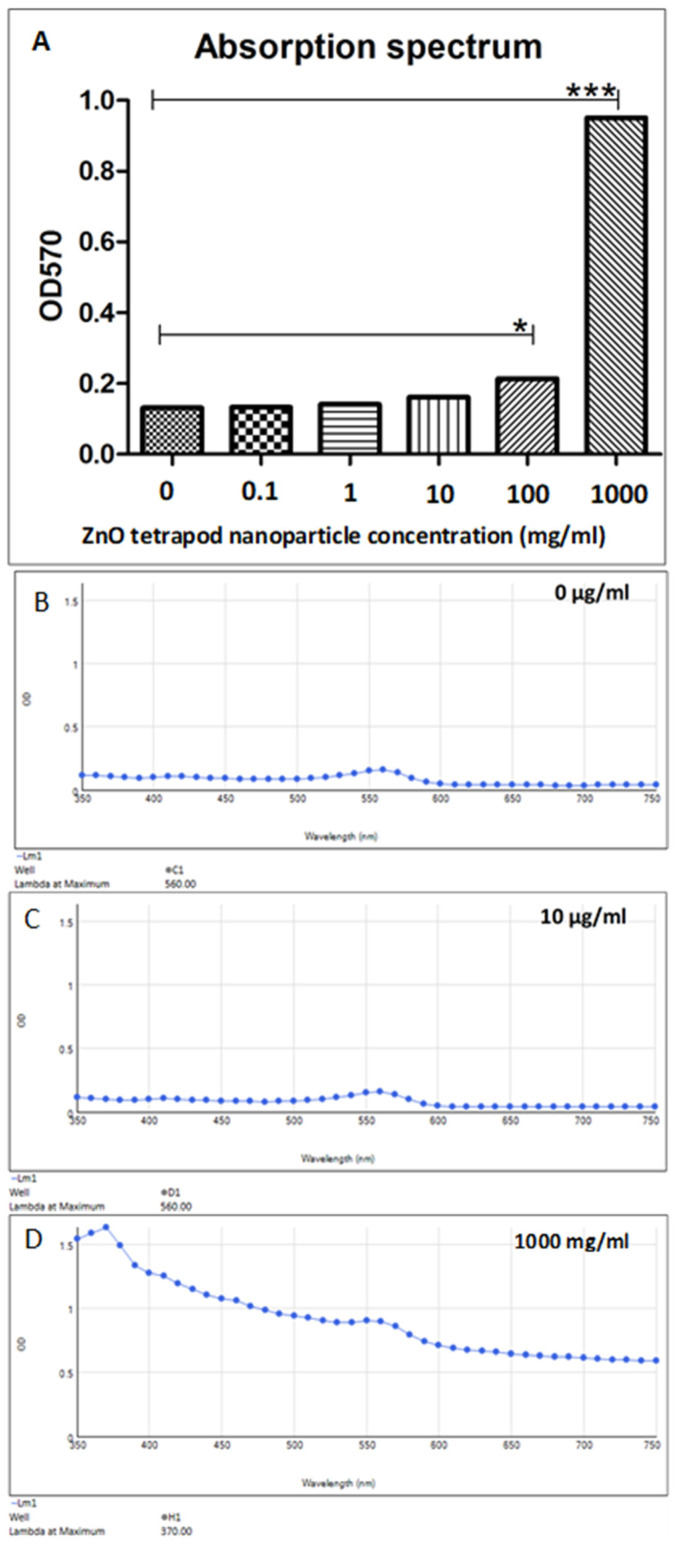
Absorption spectra of different ZnO-T concentrations at 570 nm. (**A**) Statistical analysis shows a significant increase in absorption at ZnO-T concentrations of 0 and 1000 mg/mL compared to untreated culture medium (*p* < 0.05 (*) and <0.001 (***), one-way ANOVA, Dunn’s Multiple Comparison Test). (**B**–**D**) Absorption spectra of 0 µg/mL, 10 µg/mL, and 1000 mg/mL.

There was no significant difference in the absorption spectrum of ZnO-T at low concentrations (0–10 µg/mL). A significant increase in the absorption was detected at higher concentrations of 100 and 1000 mg/mL ZnO-T (*p* < 0.05 and <0.001, one-way ANOVA, Dunn’s Multiple Comparison Test) (Figure 2). This step was necessary to test whether the physical presence of ZnO-T could influence the MTT.

### 3.3. Cytotoxicity of ZnO-Tetrapods

The cytotoxic potential of the ZnO-tetrapods was first investigated more closely using HTF cell cultures.

**Figure 3:**
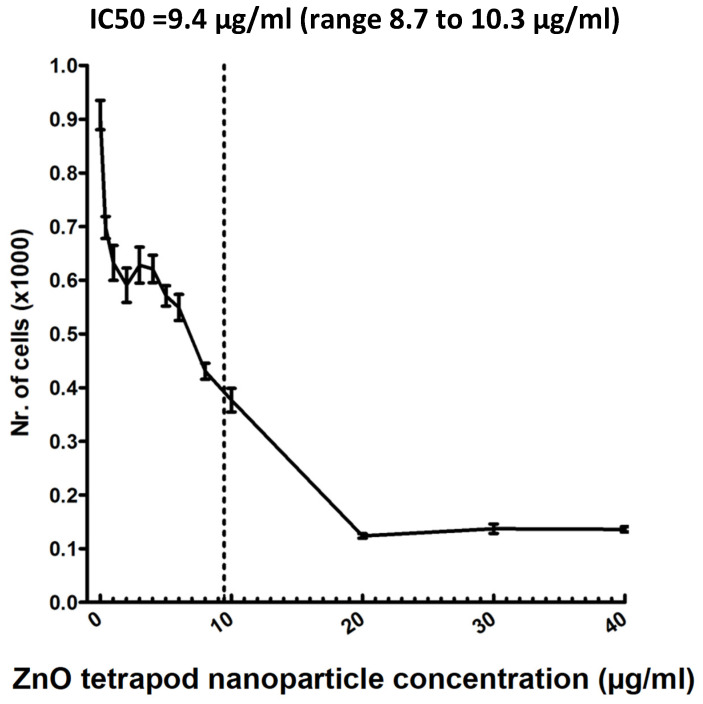
MTT test: ZnO-T toxicity was tested in a human Tenon’s fibroblast culture. The half maximal inhibitory concentration (IC50) was 9.4 µg/mL (range: 8.7 to 10.3 µg/mL).

To estimate the IC50, a four-parameter logistic nonlinear regression model was used (Figure 3). Using the MTT test, cell viability was revealed at an IC50 of 9.4 µg/mL (range 8.7–10.3 µg/mL). 

### 3.4. Ki67, α-SMA and pSMAD Immunostaining

**Figure 4:**
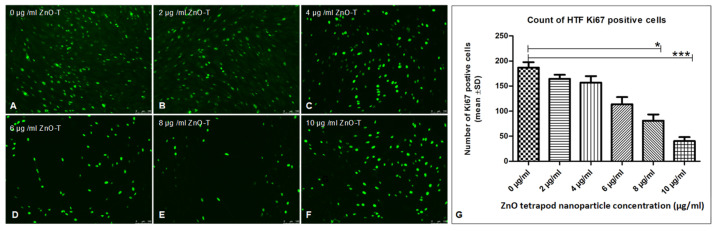
Effect of ZnO-T on human Tenon’s fibroblast (HTF) proliferation: (**A**) The control (medium only) shows a high Ki67 expression (green dots represent nuclei of proliferating HTFs). (**B**–**F**) Treatment with ZnO-T results in a reduction of Ki67 expression in HTFs, indicating a reduction in cell proliferation. (**G**) Statistical analysis shows a significant reduction of HTF proliferation with concentrations of 8 and 10 µg/mL ZnO-T in the medium compared to the control (*p* < 0.05 (*) and <0.001 (***), one-way ANOVA, Dunn’s multiple comparison Test).

**Figure 5:**
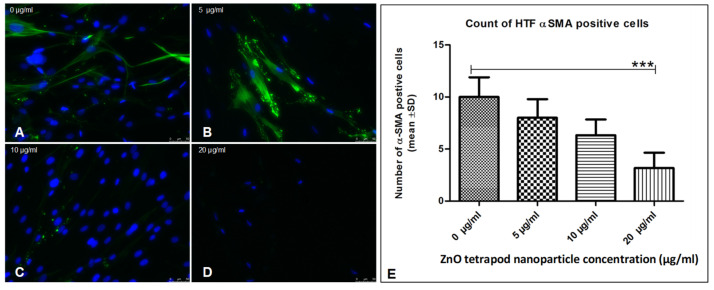
Effect of ZnO-T on human Tenon’s fibroblast (HTF) contractility (α-SMA expression): (**A**) HTFs of the control group (medium only) exhibited an intense fibrillar pattern of α-SMA-specific proteins. Blue = nuclei, Green = α-SMA-positive fibrils. (**B**–**D**) Treatment with ZnO-T results in a reduction of α-SMA expression in HTFs, indicating a reduction in cell contractility. (**E**) Statistical analysis shows a significant reduction of HTF contractility at a concentration of 20 µg/mL ZnO-T in the medium compared to the control (*p* < 0.001 (***), one-way ANOVA, Dunn’s multiple comparison Test).

**Figure 6:**
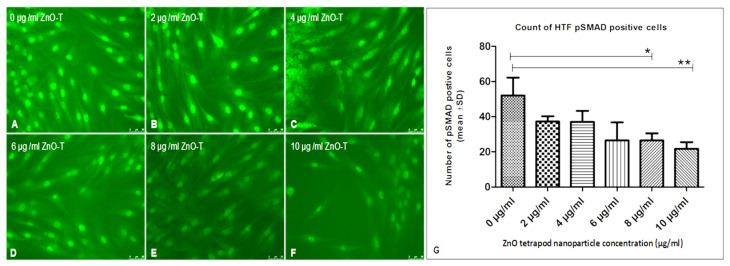
Effect of ZnO-T on human Tenon’s fibroblast (HTF) transdifferentiation (*p*-SMAD expression): (**A**) HTFs of the control group (medium only) showed many *p*-Smad-positive cells (**B**) Treatment of HTF with ZnO-T results in a reduction of *p*-SMAD expression, indicating decreased cell transdifferentiation (**B**–**F**). (**G**) Statistical analysis shows a significant reduction of HTF transdifferentiation at concentrations of 8 and 10 µg/mL ZnO-T (**E**,**F**) compared to the control (*p* < 0.05 (*), *p* < 0.01 (**), One-way ANOVA, Dunn’s multiple comparison Test).

Using Ki67 staining, a significant antiproliferative effect was shown after 6 h of treatment with 8 and 10 µg/mL ZnO-T compared to control cells (*p* < 0.05 and <0.001, one way ANOVA, Dunn’s Multiple Comparison Test) (Figure 4). α-SMA-positive cell count as a marker for fibroblast contractility was significantly reduced after treatment with 20 µg/mL ZnO-T compared to controls (*p* < 0.001, one-way ANOVA, Dunn’s Multiple Comparison Test) (Figure 5). pSMAD-positive cells as a marker for transdifferentiation were significantly reduced after treatment with 8 and 10 µg/mL ZnO-T compared to the controls (*p* < 0.05, *p* < 0.01, one-way ANOVA, Dunn’s Multiple Comparison Test) (Figure 6).

### 3.5. Cell Migration: Wound Healing Assay

**Figure 7:**
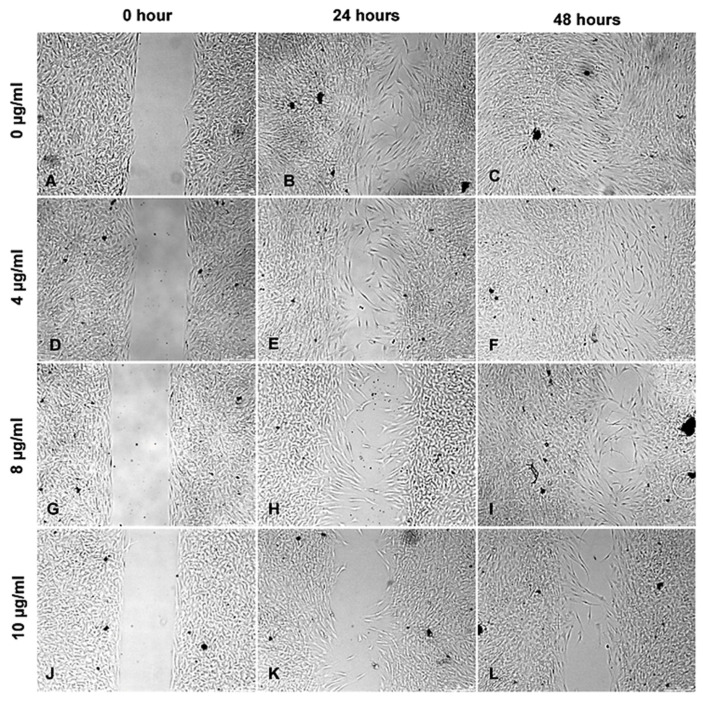
Wound healing assay following treatment with ZnO-T for 6 h: Phase contrast photomicrographs of human Tenon’s fibroblasts (HTF) show a concentration and time-dependent inhibition of HTF migration and proliferation following treatment with ZnO-T at 4–10 µg/mL (**D**–**L**) compared to untreated cells (**A**–**C**). The antiproliferative effect continues after cessation of the 6 h treatment within the next 24 (**E**,**H**,**K**) and 48 h (**F**,**I**,**L**). The cells show no toxic changes after incubation with the highest concentration of 10 µg/mL ZnO-T for 6 h (**K**,**L**).

**Figure 8:**
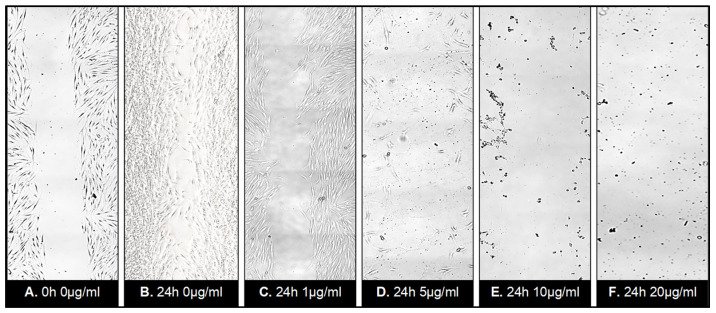
Wound healing assay following treatment with ZnO-T for 24 h: Phase contrast photomicrographs of human Tenon’s fibroblasts (HTF) in a wound healing assay. The rectangle (**A**) represents the double wound gap area at baseline. At 24 h, the assay shows migration and proliferation in control assay at 24 h (B). In contrast, a reduction in migration and proliferation following incubation with ZnO-T (1 µg/mL/24 h) (**C**) compared to controls (**A**,**B**). Treatment for 24 h with 5 µg ZnO-T and more causes concentration-dependent toxic effects, manifested as a loss of HTF at the wound edges (**D**–**F**).

**Figure 9:**
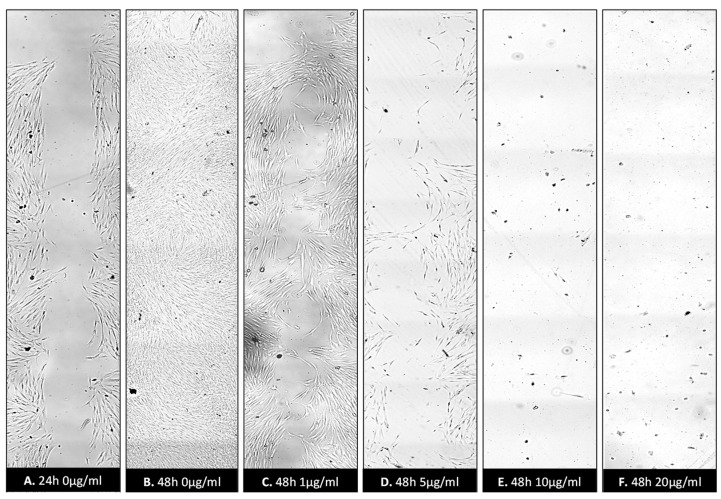
Wound healing assay following treatment with ZnO-T for 48 h: Inhibition of HTF migration and proliferation was observed following treatment with ZnO-T at concentration range of (1–20 µg/mL/48 h)**.** Control assay (**A**) appears completely healed at 48 h (**B**). HTF treated with 1 and 5 µg/mL ZnO-T appears in good condition (**C**,**D**). However, their numbers are notably reduced (**D**). Treatment for 48 h caused toxic effects manifested as loss of HTFs at the wound edges. At 10 µg/mL and 20 µg/mL, only a few cells survived (**E**,**F**).

**Figure 10:**
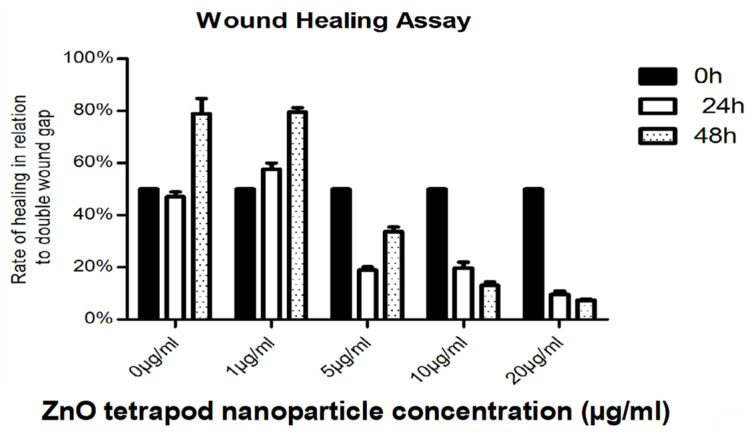
Wound healing rates at 24 and 48 h post-treatment: The cell-free wound area at 24 and 48 h was correlated to the initial double wound area (to consider lost cells at the wound margins). Lower proliferation rates at 48 h after treatment with 10 and 20 µg ml ZnO-T are explained by toxic effects.

**Figure 11:**
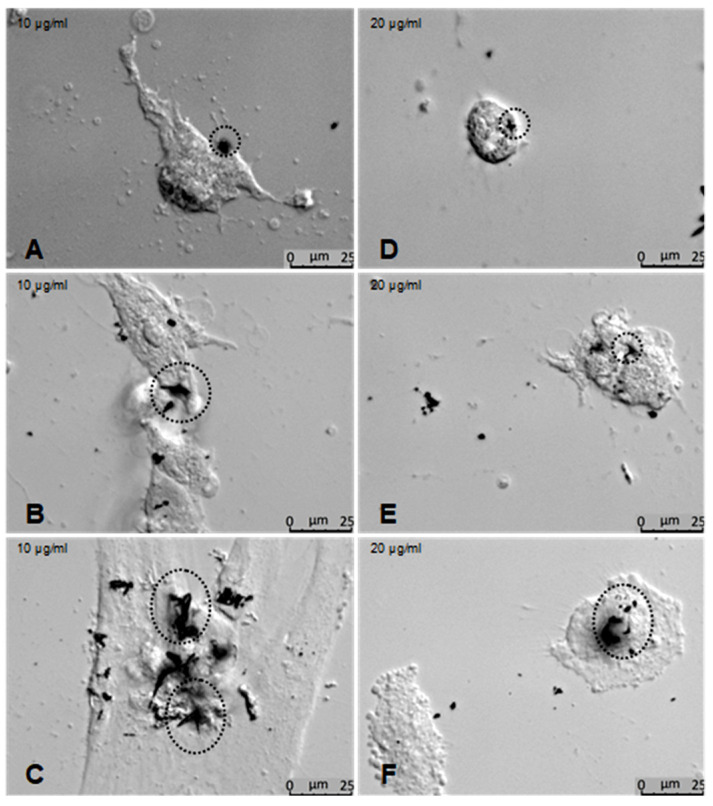
Effects of ZnO-T on human Tenon’s fibroblasts (HTF) at high concentrations: (**A**–**C**) Phase contrast photomicrographs of HTFs exposed to 10 µg/mL ZnO-T for 48 h showed morphological changes that varied from loss of the spindle shape to reduction of the cytoplasm to cell death. ZnO-T particles were detected at the cell surface (circles). At a concentration of 20 µg/mL for 48 h, the cells acquired a rounded shape (**D**–**F**).

Application of ZnO-T for 6 h was effective at relatively higher doses (10 µg/mL/6 h). With prolonged treatment, lower doses (5 µg/mL/48 h) inhibited migration significantly, while cell toxicity became more apparent at higher doses (10µg/mL/48 h). Without treatment, fibroblasts covered the scratched area within 24 h. Migration of HTF was significantly reduced after ZnO-T treatment (10 µg/mL/6 h). This migration-inhibiting effect persisted for up to 48 h after treatment cessation, with no cell toxicity (Figure 7). Significant reduction in cell migration compared to the control was observed after 24 and 48 h of treatment with 5, 10, and 20 µg/mL ZnO-T (*p* < 0.001, two-way ANOVA, Bonferroni post-test) (Figure 8, Figure 9 and Figure 10). However, prolonged incubation with high ZnO-T concentrations was associated with morphological changes in HTFs that varied from loss of the spindle shape to reduction of the cytoplasm to cell death. ZnO-T on the cell surface was deeply fixed in the cell membrane (Figure 11).

### 3.6. Cytokine Production: Immunoassays

**Figure 12:**
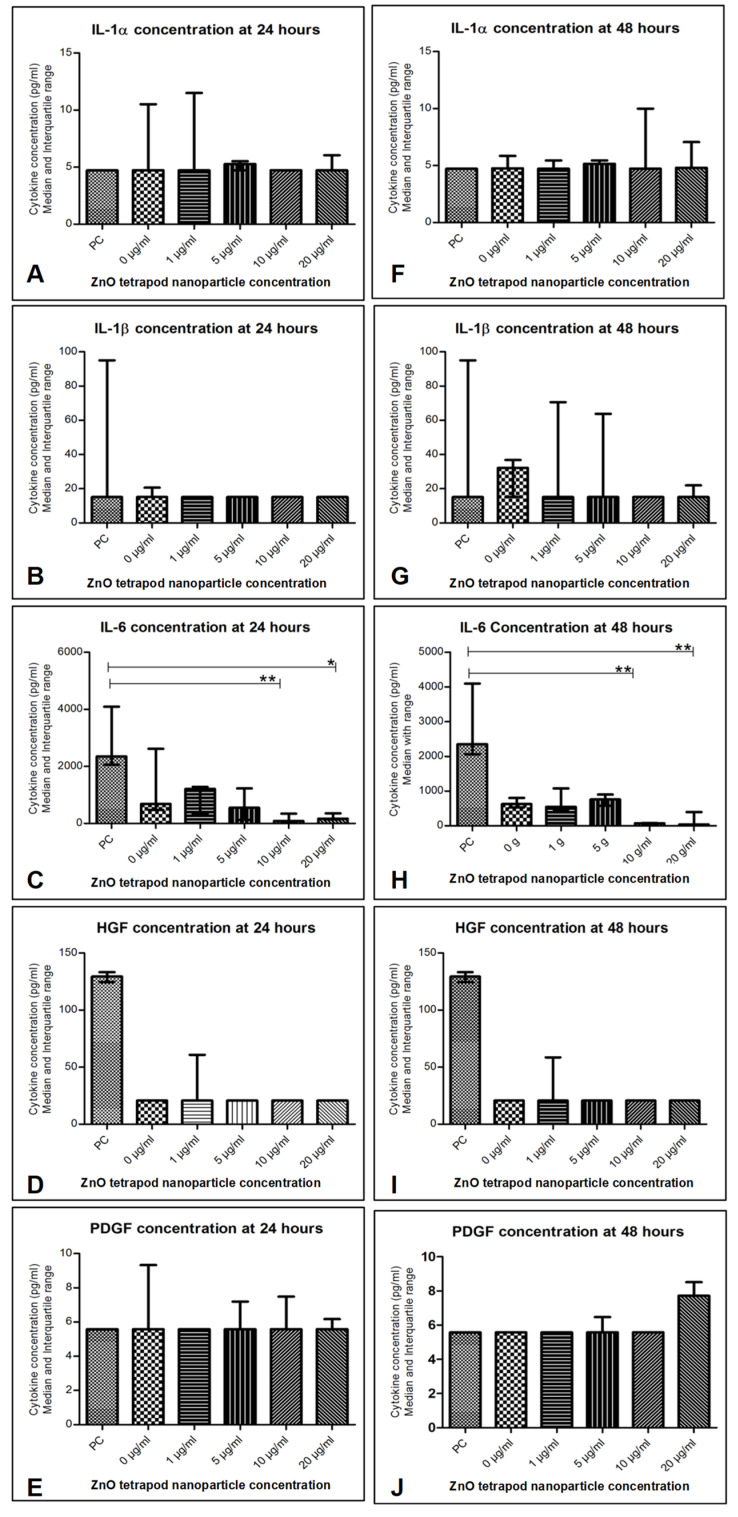
Cytokine levels in human Tenon’s fibroblasts (HTF) after treatment with ZnO-T: (**A**–**E**) 24 h following incubation with ZnO-T IL-6 was significantly reduced compared to pre-treatment (PC). This effect was significant at concentrations of 10 and 20 µg/mL (**E**). (**F**–**J**) 48 h following incubation with ZnO-T IL-6 was significantly lowered compared to pre-treatment. (*p* < 0.05 (*), *p* < 0.01 (**), One-way ANOVA, Dunn’s multiple comparison Test).

**Figure 13:**
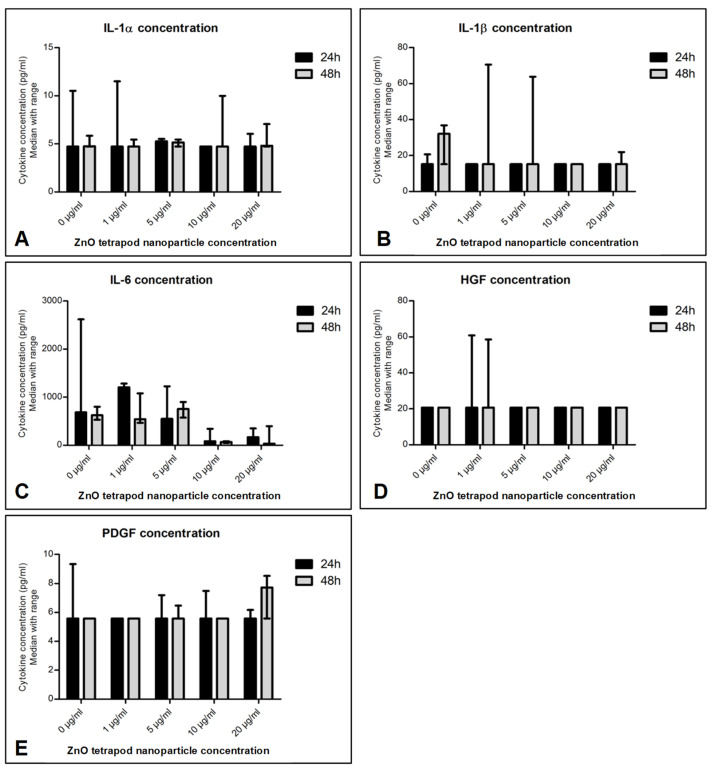
Effect of ZnO-T treatment time on cytokine production: No significant difference in cytokine secretion (**A**–**E**) was detected when comparing 24 h of incubation with ZnO-T to 48 h incubation.

IL-6 concentration was significantly reduced in culture supernatant treated with 10 and 20 µg/mL ZnO-T for 24 and 48 h compared to pre-treatment (*p* < 0.001 and <0.05, respectively, Dunn’s Multiple Comparison Test, Figure 12). There was no significant difference between the cytokine concentrations at 24 and 48 h following ZnO-T treatment (Figure 13).

## 4. Discussion

In our study, we investigated the effects of Zinc Oxide Tetrapods (ZnO-T) on primary cultures of human Tenon’s fibroblasts. We showed that ZnO-T effectively inhibited HTF proliferation, migration, and transdifferentiation. The antifibrogenic and anti-inflammatory properties of ZnO-T were demonstrated as a suppressed expression of Ki67, α-SMA, and pSMAD, as well as a reduced synthesis of the cytokines IL-6 and HGF.

The success of glaucoma filtering surgery has been improved since the introduction of cytostatic drugs, such as 5-fluorouracil [39] and mitomycin-C (MMC) [40,41,42]. However, due to their nonspecific mechanisms of action [43], sight-threatening complications may result [44]. For a better safety profile, a number of substances, e.g., trastuzumab [45], were tested in the recent past. Our group showed the wound-modulating effects of TGF-β and p38 MAPK inhibitors [46,47].

Nanotechnology has the potential to get involved in effective wound modulation following glaucoma surgery [24]. An LDL-MMC-chitosan nanoparticle drug delivery system was proposed to increase the safety and effectiveness of MMC [27]. Likewise, Ye and colleagues developed cationic nanocopolymers that improved the surgical outcome in a monkey model of trabeculectomy [26]. In our study, we examined a new anti-scarring agent at nanoscale: Zinc Oxide Tetrapods. ZnO nanoparticles (ZnO NPs) are among the most widely used nanomaterials in biomedicine [28,48,49] and were recently described as selective killers for rapidly proliferating cells, whereas differentiated cells were not affected [34]. Therefore, the HTFs, which proliferate after glaucoma filtering surgery, might also be a target for the ZnO nanoparticles. Nevertheless, ZnO nanoparticles might also be cytotoxic for the surrounding, non-proliferating tissue, which could cause a breakdown of the conjunctiva followed by postoperative hypotony. Several studies show the negative impact of nanoparticles, especially ZnO nanoparticles, which might accidentally get into the environment, undergo several bio/geo-transformations, and change their cytotoxic potential [50,51,52]. Furthermore, free Zn^2+^ ions may be released by the later-implanted material and accompany increased reactive oxygen species and endoplasmic reticulum stress [53].

Therefore, to avoid these side effects, the tetrapod structure was used. ZnO tetrapods have less cytotoxic potential than spherical ZnO nanoparticles [25]. Furthermore, they exhibit their cytotoxic effect through direct cell contact and only to a small extent through free zinc ions (16), which might be useful for a more localized antiproliferative effect and fewer side effects. The tetrapod structure of the ZnO NPs that we used in our study consisted of a ZnO core in a zinc-blended structure from which four ZnO arms radiated out of a wurtzite structure [54,55,56]. This large, biologically active structure prevents cellular uptake and maintains the nano-specific properties of the tetrapod tips [36]. Nevertheless, further studies should prove the biocompatibility and safety of using ZnO-T.

In our study, we showed that the reported MTT results were not affected by the presence of ZnO-T at tested concentrations (0–10 µg/mL). A concentration higher than 100 mg/mL can affect the MTT results, as it can interfere with the spectrophotometer absorbance quantification.

We investigated the effects of ZnO-T on primary cultures of human Tenon’s fibroblasts (HTF). HTF migration was tested using a wound-healing assay with a cell insert [57]. The HTFs were allowed to migrate freely following the removal of the cell insert. Compared to the wound scratch assay [58], this method provided a reproducible wound of fixed dimensions. Our results showed that both short- (6 h) and long-term (24 and 48 h) treatments with ZnO-T inhibited the growth and migration of the cells in a dose-dependent manner. The IC50 of 9.4 µg/mL comports with the results of a previous study, where a reduction in cell viability of proliferating cells (neuroblastoma cells) was also seen with doses higher than 10 µg/mL [59]. In detail, our experiments showed that fibroblasts tolerated treatment with concentrations of ZnO-T as high as 10 µg/mL for 6 h without toxic effects, while long-term exposure of the cells led to a significant decrease in cell count, even at the low concentration of 5 µg/mL. Thus, a brief exposure of the scleral flap and conjunctiva to ZnO-T during glaucoma surgery may be a suitable option to deliver high concentrations of ZnO-T. Extended-release systems or coated implants may be suitable to use for lower concentrations of tetrapods to suppress wound healing. Prolonged treatment at high concentrations of ZnO-T (10 and 20 µg/mL) was associated with morphological changes in the fibroblasts, such as losing their spindle shape or reducing their cytoplasm. As cellular uptake of the tetrapods could be excluded [36], it is possible that the toxicity occurred due to a disruption of the cell membrane by the tips of the ZnO-T. An aggregation of ZnO-T was seen in the nuclear zone of treated cells.

The expression of Ki67, SMA, and pSMAD in vitro following short-term treatment with ZnO-T for 6 h was sufficient to suppress fibroblast proliferation, migration, and mesenchymal transformation functions. This might indicate a potential long antifibrotic effect without the need for repeated treatment.

The antifibrotic effect of ZnO-T was manifested in our study as an inhibition of HTF migration, proliferation, and transdifferentiation. Furthermore, the concentration of Human Growth Factor (HGF) in culture-supernatant samples was lower compared to the controls. HGF stimulates cell proliferation, motility, morphogenesis, and angiogenesis in various organs [60].

Lower IL-6 concentrations in culture supernatants with ZnO-T treatment indicate an anti-inflammatory effect of ZnO-T. IL-6 is a pleiotropic cytokine that is involved in the growth and differentiation of numerous cell types [61]. ZnO-T suppresses IL-6 production by HTFs even at low concentrations of 1 µg/mL. This drop in IL-6 may reflect the antiproliferative abilities of ZnO-T but may also be associated with potential toxic effects at higher concentrations. The low IL-6 concentration might also reflect a mild inflammatory response following treatment with ZnO-T. Previous studies have already revealed an antibacterial effect of ZnO nanoparticles, such as, for example, on Staphylococcus aureus or Streptococcus agalactiae [62,63]. Staphylococcus and Streptococcus species play the most important role in postoperative bleb infection and endophthalmitis [64,65]. Therefore, ZnO tetrapods might not only have a local antiproliferative effect, thus minimizing postoperative fibrosis, but also reduce the risk of postoperative infections.

In order to overcome the poor solubility of ZnO-T, we diluted them in large volumes, mixed them repeatedly in a vortexer, and performed multiple experiments. Although the wound assay with the insert plate did not represent the tissue response after injury exactly, it provided optimal conditions to understand cell migration and proliferation. Nevertheless, as mentioned above, we should keep in mind that better solubility might increase the toxicity of the ZnO-T [66], even though we did not notice it in our experiments.

Speaking of a new glaucoma implant material, we need to consider that ZnO-T, in combination with another implant material, might also change the cytotoxic effect or the efficacy of ZnO-T material. Furthermore, both of these materials might be influenced by the natural human environment of the HTFs, as our experiments are not influenced by the aqueous humor and the natural cytokine levels [67].

In conclusion, this study shows that ZnO tetrapods inhibit wound-healing processes such as fibroblast proliferation, migration, transdifferentiation, and cytokine release. Therefore, ZnO-T may represent an innovative approach for wound healing modulation in ocular surgery or serve as a coating material for ocular implants. Nevertheless, further studies are necessary before human application, especially to prove the safety of such material.

## 5. Patents

The application process has been started.

## Data Availability

Data underlying the results presented in this paper are not publicly available at this time but may be obtained from the authors upon reasonable request.
